# Mini Review: Virus Interference: History, Types and Occurrence in Crustaceans

**DOI:** 10.3389/fimmu.2021.674216

**Published:** 2021-06-11

**Authors:** César Marcial Escobedo-Bonilla

**Affiliations:** Laboratory of Pathology and Molecular Diagnostics, Aquaculture Department, Instituto Politécnico Nacional - CIIDIR Unidad Sinaloa, Guasave, Mexico

**Keywords:** virus interference, tissue cultures, cell cultures, bacteria hosts, vertebrate hosts, crustacean hosts

## Abstract

Virus interference is a phenomenon in which two viruses interact within a host, affecting the outcome of infection of at least one of such viruses. The effect of this event was first observed in the XVIII century and it was first recorded even before virology was recognized as a distinct science from microbiology. Studies on virus interference were mostly done in the decades between 1930 and 1960 in viruses infecting bacteria and different vertebrates. The systems included *in vivo* experiments and later, more refined assays were done using tissue and cell cultures. Many viruses involved in interference are pathogenic to humans or to economically important animals. Thus the phenomenon may be relevant to medicine and to animal production due to the possibility to use it as alternative to chemical therapies against virus infections to reduce the severity of disease/mortality caused by a superinfecting virus. Virus interference is defined as the host resistance to a superinfection caused by a pathogenic virus causing obvious signs of disease and/or mortality due to the action of an interfering virus abrogating the replication of the former virus. Different degrees of inhibition of the superinfecting virus can occur. Due to the emergence of novel pathogenic viruses in recent years, virus interference has recently been revisited using different pathogens and hosts, including commercially important farmed aquatic species. Here, some highly pathogenic viruses affecting farmed crustaceans can be affected by interference with other viruses. This review presents data on the history of virus interference in hosts including bacteria and animals, with emphasis on the known cases of virus interference in crustacean hosts.

Life Science Identifiers (LSIDs)

*Escherichia coli* [(Migula 1895) Castellani & Chalmers 1919]

*Aedes albopictus* (Skuse 1894)

*Liocarcinus depurator* (Linnaeus 1758): urn:lsid:marinespecies.org:taxname:107387

*Penaeus duorarum* (Burkenroad 1939): urn:lsid:marinespecies.org:taxname:158334

*Carcinus maenas* (Linnaeus 1758): urn:lsid:marinespecies.org:taxname:107381

*Macrobrachium rosenbergii* (De Man 1879): urn:lsid:marinespecies.org:taxname:220137

*Penaeus vannamei* (Boone 1931): urn:lsid:zoobank.org:pub:C30A0A50-E309-4E24-851D-01CF94D97F23

*Penaeus monodon* (Fabricius 1798): urn:lsid:zoobank.org:act:3DD50D8B-01C2-48A7-B80D-9D9DD2E6F7AD

*Penaeus stylirostris* (Stimpson 1874): urn:lsid:marinespecies.org:taxname:584982

## Introduction

Virus interference is a phenomenon observed before the dawn of virology itself and even before proper experimental methods were available in the decade of 1950 (1, 2). The earliest records indicating the presence of interference between two pathogens go back to the XVI century when Montaigne observed that one disease could be cured by another. In the XVIII century it was reported that the severity of smallpox infection in children was reduced when they had secondary yaws. In the XIX century, cowpox vaccination prevented the full manifestation of smallpox infection. Also, vaccination reduced symptoms of whooping cough in children (3).

Virus interference was first described in plants in 1929 by McKinney where the yellow-mosaic tobacco virus did not replicate in plants already infected with the common mosaic virus (4). In animals, the first observations of virus interference were done in 1935 by Magrassi studying two strains of herpesvirus in rabbit with different tissue tropism. Rabbits infected with non-encephalitogenic strains of herpesvirus were resistant to infection by an encephalitogenic strain inoculated in the brain. The same year, a study done by Hoskins with strains of yellow fever virus with different tropism showed that monkeys infected with a neurotropic strain were protected against a lethal infection with a pantropic strain (4, 5). Another study done in 1937 by Findlay and MacCallum with the Rift Valley fever virus in rhesus monkeys, protected them from an infection with the unrelated yellow fever virus (5–7). In viruses infecting bacteria (bacteriophages), the first virus interference reports were done in 1942 (8, 9). Here, a strain of *Escherichia coli* [(Migula 1895) Castellani & Chalmers 1919], was used as host upon which two distinct bacteriophage strains (by replication speed and size of inhibition halo produced), were able to replicate. The interference experiments showed that using equal amounts of these two viruses (α and γ), virus γ inhibited replication of virus α by 67%. Also, the interference did not occur by differences in virus adsorption by the cells, and the time at which virus γ was inoculated after virus α influenced the interference, with longer times (4 - 6 min) showing less interference (8). Since these earliest works, subsequent records of virus interference have been documented between homologous and heterologous viruses in human cells as well as in animal hosts.

Different *in vitro* and *in vivo* systems have been used to study virus-virus interactions resulting in interference. These have included avian chorio-allantoic membranes (1, 10), prokaryotic (8, 9) and eukaryotic cell cultures (4, 11), and animal hosts such as mosquitoes (1), insect larvae (12), mice, rats, hamsters, rabbits, horses, primates, including man (1, 4, 7), ferrets (13), and more recently, fish (14) and crustaceans (15). Fish and shellfish are appreciated food commodities with significant commercial value and are produced in aquaculture facilities throughout the world (16). These studies show that under natural conditions, virus coinfections often occur within a single host (17–22) and virus replication of the coinfecting viruses can occur within the same or different cellular compartments (1, 3, 17).

Virus interference is a virus-virus interaction in which the infection and/or replication of one virus is altered by the presence of another virus within the same host (4, 5, 7, 8, 19). Such interaction can occur *in vivo* or *in vitro*. This type of virus interactions may affect the pathogenesis of at least one of the viruses (18, 19). Most of the knowledge on quantitative interference derives from work on myxoviruses in chick embryos or on tissue cultures. Moreover, interference reported in embryonated eggs or in tissue cultures does not involve antibody production, therefore ensuring that the virus interference is independent of immune responses (1).

### Interference Experiments *In Vivo*

The *in vivo* interference experiments in monkeys were first done in 1935 and 1937 (4–7). The *in vivo* interference assays in vertebrates may be covert by the presence of immune defense responses and the production of molecules that may be confused with virus interference. Such molecules include antibodies, production of innate immune responses, interferons (IFNs) and other cytokines, cellular immune responses or immunostimulation (1, 5, 10, 19, 23). Also, some interference experiments may have induced the genetic recombination of less virulent virus strains, producing a mock interference result (1, 19).

Despite these drawbacks, the virus interference phenomenon has been reported *in vivo* using various vertebrate hosts such as mice, guinea pigs, hamsters, rabbits, foxes, hedgehogs, ferrets, goats, calves, chick embryos, chickens and non-human primates (1, 3, 6, 13, 24–26) (**Table 1**). In all these cases, the interference was due to virus components and not to any innate or specific host immune response (1, 13). Examples of this are described in Types of Virus Interference. A study showed that production of virus-specific IFN-γ was not the cause of the observed interference in heterotypic viruses. Lymph nodes of ferrets infected with influenza B virus showed IFN-γ responses to this virus but not to Influenza A (H3N2) virus and vice versa; ferrets infected with Influenza A (H3N2) virus had IFN-γ responses specific to this virus but not to Influenza B virus (26). The *in vivo* experiments found that one particle of interfering virus was enough to cause interference of the superinfective virus in different homologous and heterologous systems (1, 3, 23, 27).

**Table 1:** Virus interference in different animal hosts using different experimental systems.

Experimental system	Host	Interfering virus	Superinfective virus	Reference
Cell culture	*Escherichia coli*	Bacteriophage T2 “γ”	Bacteriophage T1 “α”	(8)
Cell culture	*Escherichia coli*	Bacteriophage “T2 “γ”	Bacteriophage T1 “α”	(9)
Cell culture	*Escherichia coli*	Bacteriophage T7 “δ”	Bacteriophage T1 “α”	(18)
Tissue culture	chick embryo tissues	Influenza A strain W.S.	Influenza A neurotropic variant “neuroflu”	(7)
Tissue culture	Chick embryo	Yellow fever virus strain 17DD	Yellow fever virus strain Asibi	(4)
Tissue culture	Chick embryo	Yellow fever virus strain 17DD	West Nile virus	(4)
Tissue culture	Chick embryo	Yellow fever virus strain 17DD	Venezuelan equine encephalomyelitis virus	(4)
Tissue culture	Chick embryo	West-Nile virus	Venezuelan equine encephalomyelitis virus	(4)
Tissue culture	Chick embryo	Yellow fever virus strain 17DD	Influenza A virus strain PR8	(4)
Tissue culture	Chick chorio-allantoic membrane	Influenza A virus	Heterologous Influenza	(1)
Tissue culture	Mouse lung	Influenza A virus	Heterologous Influenza	(1)
Tissue culture	Chick chorio-allantoic membrane	Influenza A virus	Newcastle disease virus	(1)
Tissue culture	Chick chorio-allantoic membrane	Influenza A virus	Mumps	(1)
Tissue culture	Chick chorio-allantoic membrane	Influenza A virus	Venezuelan equine encephalomyelitis virus	(1)
Tissue culture	Chick chorio-allantoic membrane	Newcastle disease virus	Influenza A virus	(1)
Cell culture	Chick embryo cells	Vesicular stomatitis virus New Jersey strain	Vesicular stomatitis virus Indiana strain	(27)
Tissue culture	Primary rabbit kidney cells	Rubella virus	Vaccinia virus	(11)
Tissue culture	Primary rabbit kidney cells	Rubella virus	Vesicular stomatitis virus	(11)
Cell culture	Rabbit kidney cell line PK13	Rubella virus	Vaccinia virus	(11)
Cell culture	Rabbit kidney cell line PK13	Rubella virus	Vesicular stomatitis virus	(11)
Cell culture	Vero-Green Monkey kidney cells	Vesicular stomatitis virus	Vesicular stomatitis virus	(26)
Cell culture	293T human cells	Human parainfluenza 3 virus	Homologous virus	(28)
Tissue culture	Chick embryo	Avian influenza virus	Newcastle disease virus	(29)
Cell culture	C6/36 *Aedes albopictus* cells	Sindbis virus	Dengue virus	(30)
Cell culture	BHK-21 cells	Pest des petits ruminants virus	Foot and mouth disease virus	(24)
Cell culture	Vero cells	Foot and mouth disease virus	Pest des petits ruminants virus	(24)
*In vivo*	Rhesus monkey	Yellow fever virus, neurotropic strain	Yellow fever virus, viscerotropic strain	Hoskins 1935 (4)
*In vivo*	Rabbit	Non-encephalitogenic Herpes simplex virus	Encephalitogenic herpes simplex virus	Magrassi 1935 (4)
*In vivo*	Rhesus monkey	Rift Valley fever virus	Yellow fever virus	(6)
*In vivo*	Mouse	Rift Valley fever virus	Yellow fever virus	(6)
*In vivo*	Mouse	Coxsackie	Poliomyelitis virus	(1)
*In vivo*	Rat	Saint Louis encephalitis	Eastern equine encephalomyelitis virus	(1)
*In vivo*	Rat	Japanese B encephalitis	Eastern equine encephalomyelitis virus	(1)
*In vivo*	Rabbit	Western equine encephalomyelitis virus	Eastern equine encephalomyelitis virus	(1)
*In vivo*	Guinea pigs	Western equine encephalomyelitis virus	Eastern equine encephalomyelitis virus	(1)
*In vivo*	Man	Dengue virus	Yellow fever virus	(1)
*In vivo*	Mosquito	Dengue virus	Yellow fever virus	(1)
*In vivo*	Chick embryo	Influenza A virus	Western equine encephalomyelitis virus	(25)
*In vivo*	Ferret	Influenza A virus (H_1_N_1_)	Influenza B virus	(31)
*In vivo*	Ferret	Influenza A virus (H_1_N_1_)	Influenza A virus (H_3_N_2_)	(31)
*In vivo*	Ferret	Influenza A virus (H_3_N_2_)	Influenza A virus (H_1_N_1_)	(31)
*In vivo*	Ferret	Influenza B/Malaysia (B/Vic) virus	Influenza B/Florida (B/Yam) virus	(13)
*In vivo*	Ferret	Influenza B/Florida (B/Yam) virus	Influenza B/Malaysia (B/Vic) virus	(13)
*In vivo*	Ferret	Influenza B/Brisbane (B/Vic) virus	Influenza B/Massachusetts (B/Yam) virus 3	(13)
*In vivo*	Ferret	Influenza B/Massachusetts (B/Yam) virus	Influenza B/Brisbane (B/Vic) virus	(13)
*In vivo*	Ferret	Influenza B/Brisbane (B/Vic) virus	Influenza B/Phuket (B/Yam) virus	(13)
*In vivo*	Ferret	Influenza B/Phuket (B/Yam) virus	Influenza B/Brisbane (B/Vic) virus	(13)
*In vivo*	Shrimp	Taura syndrome virus	Yellow head virus	(32)
*In vivo*	Shrimp	Infectious hypodermal and haematopoietic necrosis virus	White spot syndrome virus	(15, 33–35)
*In vitro*	Shrimp	Infectious hypodermal and haematopoietic necrosis virus	White spot syndrome virus	(36)
*In vivo*	Crab	Unknown virus	Unknown virus	(37)

### Interference Experiments *In Vitro*

Virus interference was first studied in 1939 using animal cell and tissue cultures with different viruses (4, 7). Cell and tissue cultures represent a more standardized and easier way to determine virus interference than *in vivo* experiments with animals (4). These systems were used to test the interference of various pairs of homologous (same species or immunologically related) or heterologous (different species or immunologically distinct) virus interactions resulting in interference (1, 3, 4, 17, 24). The first tissue cultures were comprised by the chick chorio-allantoic membrane (8), minced mouse embryos or chick embryos without nervous tissues (3, 4, 7). Hence, cultures were not composed by a homogeneous cell type. Nonetheless, in these types of culture systems, the interference was evaluated through the replication inhibition of the superinfecting virus by the interfering virus. Interference of the cultured viruses was additionally evaluated by the corresponding virus titers *in vivo* using susceptible hosts (3, 4, 7). Later, using modern continuous cell cultures (e.g. continuous mouse L cell cultures; rabbit kidney cell line RK13, Vero-Green monkey kidney cells), experiments were carried out to determine the interference using different animal viruses (11), showing that interference actually occurs between various types of viruses and that such an interference was not caused in tissue or cell cultures by immunologic factors such as neutralizing antibodies, humoral immunity (3, 4) or the presence of soluble antiviral molecules such as IFNs (5, 7, 10, 11, 28, 29).

In such culture systems, cells pre-infected with an interfering sublethal virus (28) or a defective interfering virus (29) become resistant to infection by a cell-destructing virus (28, 29). Moreover, experiments done in chick embryo cell cultures using vesicular stomatitis virus (VSV), showed that only one interfering virus particle is needed to block infection of a superinfecting virus. Also, similar to what was previously reported for bacteriophages (8), an all or nothing effect was observed in these experiments (27, 29). The virus interference between rubella virus and vaccinia or VSV was not caused by the presence of IFN in primary rabbit kidney cell cultures or when using an excessive amount of IFN in the continuous rabbit kidney cell line PK13 (11). In these culture systems, interference was found between homologous and heterologous viruses (**Table 1**). Examples of such studies include tests between strains of avian influenza A virus with strains of Newcastle disease virus in chicken embryos (25), and the interference of Sindbis virus inoculated 1 h before challenge with dengue virus using *Aedes albopictus* (Skuse 1894) C6/36 cell cultures (30).

## Types of Virus Interference

Different studies were done to determine virus interference in various hosts. These include bacterial cells, animal cell and tissue cultures and *in vivo* experiments (**Table 1**). Since the earliest studies on virus - virus interference, it was suggested that the interference mechanism may not be one but many (1, 3, 18, 23). Recently, different situations of virus - virus interactions were analyzed and three main categories of virus-virus interactions were proposed: (a) direct interactions of viral genes or gene products, (b) alterations in the host environment leading to indirect virus interactions, and (c) immunological interactions occurring only in animals with adaptive immune systems (19). This classification of virus-virus interactions do not deal with virus interference but rather the possible outcomes of such interactions. Nonetheless, the first category includes the main type of the virus interference phenomenon reported in bacterial and animal hosts.

### Direct Interactions of Viral Genes or Gene Products

This category consist of the physical interaction of nucleic acids or proteins of one virus with genes or gene products of another infecting virus. This category includes the superinfection exclusion event, which is the main example of virus interference.

#### Superinfection Exclusion

This type of virus interaction occurs when a primary viral infection induces resistance to subsequent infections by similar viruses (19). This interaction results in the most commonly reported virus interference event and it has been described among various virus types including bacteriophages, flaviviruses, orthomyxoviruses, paramyxoviruses, retroviruses, hepadnaviruses, arboviruses, and plant viruses (1, 3, 4, 18, 19). Not all the different exclusion mechanisms have been unraveled, but those known depend on direct interaction of products of the primary infection with the secondary infecting virus.

Examples of this type of virus interference include viruses infecting bacterial cells (bacteriophages). Bacteria (*E. coli*) simultaneously infected with two different types of T bacteriophages showed interference, as one single interfering virus particle was able to inhibit replication of a super-infective virus in any bacterial cell (8, 9, 18).

These experiments revealed various features on the mechanism of virus interference in bacterial cells: (i) the interference was not due to differences in virus adsorption to cells, penetration into the host cell or competition for a key enzyme (8, 18, 23); (ii) the virus interference did not induce cross-immunity (8); (iii) one cell never released both virus types. Only one type of virus was produced from any one infected cell. This was called the mutual exclusion effect (1, 18, 23); (iv) the mutual exclusion effect is an “all or none” event (8); (v) The depressor effect (1, 18, 23) results in reduced yield of both viruses produced in an infected cell, compared to their normal replication yield. Later it was proposed that both the mutual exclusion and depressor effect occurs as result of the superinfecting virus not getting access to the cell division machinery, instead of the hypothesis of the key-enzyme proposed by Delbrück and Luria (3, 18, 23). The mutual exclusion effect described in bacterial cells, may be analogous to the interference reported in some animal viruses (8, 18) given that the two viruses replicate in the same cellular compartment.

Examples of superinfection exclusion in animal hosts include various homologous interfering viruses such as influenza virus (1, 23), viscerotropic and neurotropic yellow fever virus and Theiler virus (1). Interference in homologous viruses is connected to virulence changes between two variants of the same virus. This can be evaluated by the protection of the host against the virulent variant or by determining the presence of viral progeny of the virulent strain. Here, the interfering agent always is an active virus.

The interference of homologous viruses is far more difficult to distinguish from the immunological effects and genetic interactions than for heterologous interference, since both the interfering and superinfecting viruses are antigenically equivalent (1). Nonetheless, the homologous interference can be recognized from immune responses. Studies done on homologous interference between neurotropic and viscerotropic yellow fever virus showed that the cross-protection was achieved in similar ways between the yellow fever and Rift Valley fever viruses, thus separating this effect from immune responses (1, 4, 6). In contrast, a study done by Magrassi in 1935 (4) using nonencephalitogenic and encephalitogenic herpesvirus strains, displayed the same phenomenon in neuronal pathways, but this experiment called for the possible role of antibody immunity. The difference between the yellow fever and the herpesvirus systems lie in the time interval from inoculation of the interfering and the superinfecting viruses, which was long enough to allow the stimulation of antibody-producing cells by the primary inoculum (1). Other studies showed that the dose of the superinfecting virus may have further stimulated a secondary antibody response. A study where animals were inoculated with Western Equine Encephalitis (WEE) virus after intracerebral challenge with active WEE virus, showed that the challenge virus may have become an antigen booster dose which offered protection (1).

On the other hand, most of the works done on virus interference have been done in heterologous virus systems in different hosts (1, 4, 11). This type of interference has also been reported in aquatic organisms such as crustaceans (15, 31, 38). In these studies, important information about timing and dosing of interfering and superinfective viruses was obtained, despite the fact that the interference event not always was quantified or clearly observed as inhibition of replication. Instead, interference was shown as protection against disease and/or mortality caused by the superinfecting virus (1). The degree of protection is dependent on the host-virus system and the severity of the disease leading to pathological manifestations of death, both *in vivo* or *in vitro*. Another criterion for heterologous virus interference is the lack of immunogenicity against the virus pairs by the host, or the independence of interference to any relationship to antigenic reactions and antibody production. This type of interference has been reported among viruses that are antigenically and taxonomically distinct (1).

Some experiments in animals have raised the doubt of whether viruses sharing some antigenic properties may induce reciprocal resistance. For example, the previous exposure to one type of poliovirus in monkeys or human may sensitize them in such way that vaccination with a different type activates neutralizing antibodies against all three poliovirus types. The consecutive infection with different arthropod-borne viruses which share complement-fixing (CF) and hemagglutinating (HA) antigens produces broadly cross-reacting neutralizing antibodies (1). Nonetheless, a clear distinction between specific immunity and interference as the basis for host resistance was shown by studies on Eastern Equine Encephalomyelitis (EEE) and WEE viruses (1). Mice, guinea pigs or rabbits inoculated with formalin-killed WEE virus induced long-lasting protection against intracerebral challenge of the homologous, but not the heterologous virus. The homologous challenge produced a transient infection which end was associated with a type-specific antibody response (1). After this abortive infection, the animals resisted an intracerebral superinfection with massive doses of the heterologous virus. This resistance was potent for some short time periods and was not associated with anamnestic antibody response. Hence this resistance was due to virus interference (1). Influenza virus gives another example of heterologous virus interference on “minor” cross-reacting antigens and antibodies that do not offer protection against different strains, or even a single serotype. Here, the presence of protective antibodies boost rather than inhibit lingering infection. Hence this result showed that interference was the mechanism of cross protection (1).

#### Helper-Dependent Virus

This interaction is another example of the category of direct interactions of viral genes or gene products. This occurs in any virus that may lack replication ability at some degree and hence requires the gene products of another virus to produce infection. This interaction does not produce virus interference, but it allows a defective virus to successfully replicate and exit the cell. An example of this interaction occurs in the bacteriophage P4, which is able to replicate its own genome in the presence of a coinfecting phage (P2), thus providing capsid components and cell lysis (19).

In vertebrates, an adeno-associated virus (AAV) is a replication-defective parvovirus that usually requires a host cell that is coinfected with an adenovirus or herpesvirus in order to produce virions to be released from the host cell. Later, it was discovered that AAV is not completely replication defective, since genotoxic stress and other factors may also make a host cell permissive for AAV progeny production (19).

In invertebrates, this type of virus-virus interaction was reported between two RNA viruses in the freshwater prawn *Macrobrachium rosenbergii* (De Man 1879) causing muscle whitening in the abdomen and sometimes cephalothorax, resulting in massive mortalities to postlarvae (20). Animals with the white tail disease had the two viruses replicating in cytoplasm of connective tissues and muscle cells. The *Macrobrachium rosenbergii* Nodavirus (MrNV) is icosaedrical with 27 nm diameter and its genome composed by two equimolar linear single-stranded (ss) RNA molecules of ≈ 3200 and 1250 nucleotides, respectively (20). The extra-small virus (XSV) also found in diseased animals has icosahedral shape, size of 15 nm and a genomic single linear ss RNA with size ≈ 900 bases (20). *In vitro* assays in fish cell line SSN-1 showed that MrNV alone infected cells and produced cytopathic effects. Infection resulted in synthesis of capsid proteins but no genome replication was observed and virions were empty despite that virus RNA was detected by RT-PCR. Supernatant of infected cells did not cause infection nor cytopathic effect in cell cultures, suggesting a role of XSV to complete MrNV viral replication. Further assays showed that higher amounts of MrNV resulted in higher yields of both MrNV and XSV, and signs of disease only appeared at high MrNV replication rates. These data indicate the essential role of MrNV in the disease and suggest a cooperative interaction of XSV with MrNV to complete its replication (20).

### Cell Receptor Blockade

Another possible mechanism of virus interference is the cell receptor blockade (3). This mechanism postulates the adsorption of virus to cellular receptors as the first step of infection, with the subsequent destruction of the receptor by a viral enzyme. This creates a breach in the cellular defense which allows the entry of the virus into the cell (1, 3). A recent study done *in vitro* described the interference mechanism of human parainfluenza type 3. Here, the neuraminidase activity was needed to establish homologous interference in 293T cells (39).

### Autointerference

This event causes protection or reduced virus replication in hosts inoculated with large virus doses, which in smaller quantities induce disease and high replication rates. This phenomenon was described first by Pasteur on the rabies virus in rabbits, but has also been reported for other virus-host systems such as Influenza B in chick embryos, and yellow fever, dengue and Rift Valley fever in mice (1). In tissue and cell cultures, autointerference has been observed in egg-adapted influenza strains on chick embryo lung monolayers, VSV in chick embryo monolayers, WEE in mice L cells. In most virus systems, autointerference is the result of virus infection that are not completely adapted to the experimental hosts, or which have mixtures of particles with different properties (e.g. different tissue tropism, varying particle virulence or completeness). These differences may result in inhibition of replication of the virulent component and hence, host protection from infection. In influenza viruses, experimental support exists of intrinsic viral heterogeneity, and these variations are the cause of the production of incomplete virions, which induce the autointerference (1).

## Virus Interference in Crustaceans

The culture of aquatic organisms such as fish, crustaceans and mollusks has been done in countries such as China, Egypt or Italy for thousands of years (40–42), but as a science and as commercial industry, aquaculture of marine and brackishwater species is probably the most recent animal production activity (43, 44). The first viral pathogens described in wild crustaceans date back to 1966 in the crab *Liocarcinus depurator* (Linnaeus 1758) (45). Later, other viruses were found in this species, and in the marine shrimp *Penaeus duorarum* (Burkenroad 1939), the virus *Baculovirus penaei* (46) was first described (46, 47). Since its beginnings, aquaculture has been affected by several viruses which have caused important damages to the industry (48). At present, the main cultured crustaceans are marine shrimp, freshwater prawns and crabs (16). Crustaceans do not have an adaptive immune system but only an efficient innate defense system to recognize and eliminate foreign materials, including microorganisms (49). Several reports exist on the coinfection of two viruses in wild or farmed crustaceans (20–22, 37, 50) which suggests the high probability of virus-virus interactions in these hosts. In fact, a few events of virus interference have been reported in crustaceans and are presented below.

### Virus Interference in Crabs

One unidentified pathogenic virus infecting hemocytes in the crab *Carcinus maenas* (Linnaeus 1758) was first mentioned in 1971 and later found to display autointeference in the same host (51, 52). The virus caused impaired hemolymph clotting and death of infected animals. Nonetheless, a majority of animals showing disease recovered and regained clotting function, about half of them at 4 - 6 d post infection (dpi). Despite recovery, crabs still had virus in hemolymph as late as 40 dpi. Low virus dilutions did not cause signs of infection but higher dilutions (10^-2^ and 10^-3^) did. This effect is similar to the phenomenon of autointerference observed in vertebrates. Autointerference was tested with undiluted and diluted hemolymph samples and it was observed when the more diluted samples caused disease faster than the undiluted ones. All six samples of whole hemolymph taken late in the course of disease did not show the normal dilution effect or even showed a reversal. Further, none of the fresh sera samples taken showed the expected normal dilution effect, and five out of six (83%) showed autointerference. This phenomenon could not be determined to be caused by the virus, from an associated virus or by the innate defense system (52).

### Virus Interference in Marine Shrimp

Marine shrimp is an important commodity and is produced in aquaculture facilities in many countries worldwide. The main farmed species are the Pacific white shrimp *Penaeus vannamei* (Boone 1931) and the black tiger shrimp *Penaeus monodon* (Fabricius 1798) among others (53). Many virus diseases have been reported in these species and several of them have caused important epizootics with large economic losses (48). Due to the importance of these hosts, virus interference acquire relevance since it may help to reduce or impair the impact of highly pathogenic viruses. Hence, better documented cases of virus interference exist in these species.

#### Interference Between Taura Syndrome Virus and Yellow-Head Virus

*Taura syndrome virus* (TSV) has a single stranded, positive-sense linear RNA genome and replicates in cytoplasm (54). It infects epithelia and connective tissues of integument, gills, foregut and hindgut of shrimp (55) and caused massive mortalities in wild and farmed Pacific white shrimp in Latin American and Asian countries (44). *Yellow-head virus* (YHV) has a single-stranded positive sense RNA genome and also replicates in cytoplasm (56). It causes systemic infection in gills, lymphoid organ, head soft tissues, eyestalk, nerve tissues, heart, midgut, hepatopancreas, connective tissues and muscle of shrimp (32, 57). This virus still causes mortality to infected shrimp.

These viruses have similar cell tropism indicating that they may compete upon coinfection. An experimental challenge was done with specific pathogen-free shrimp inoculated first with TSV-infected tissues *per os* to produce chronically-infected shrimp. Then, after 27, 37 or 47 dpi shrimp were challenged intramuscularly with a lethal dose (10^4^ genome copies) of YHV. Results showed that virus interference occurred between TSV and YHV and survival of shrimp treated with TSV was significantly higher than YHV positive controls at the different times evaluated. The highest survival occurred when YHV was inoculated at 27 d after TSV inoculation (31). *In situ* hybridization assays showed that shrimp were infected with the two viruses but their tissue distribution was different. Presence of TSV was restricted to lymphoid organ due to its chronic stage, whereas YHV was found in cuticular epithelium, connective tissues and lymphoid organ. In this organ, TSV was mostly present in tubules while YHV was distributed in adjacent areas of the tubules, and severe necrosis by YHV was not seen in the interference shrimp (31).

#### Interference Between IHHNV and WSSV

Probably the most studied virus interference event in shrimp is between *infectious hypodermal and haematopoietic necrosis virus* (IHHNV) (38), also known as *Penstylhamaparvovirus* 1 (58), and *white spot syndrome virus* 1 (WSSV) (59). The IHHNV has a linear single-stranded DNA genome of 4.1 kilobases (60). This virus replicates in nuclei of infected cells in organs of ectodermal and mesodermal origin (61). It caused massive mortalities to farmed Pacific blue shrimp *Penaeus stylirostris* (Stimpson 1874) and disease to *P. vannamei* and *P. monodon* (61). The WSSV genome is circular, double-stranded DNA of size between 293 - 307 kilobase pairs. Its large virion (210 - 380 nm length x 70 - 167 nm width) is enveloped, non-occluded, bacilliform with a tail-like appendage at one end (62). WSSV is a systemic pathogen replicating in organs of ectodermal and mesodermal origin. This virus still is a major threat to shrimp aquaculture worldwide.

The first evidence of interference was found in experiments done in juvenile Pacific blue shrimp *P. stylirostris* (15). Shrimp were pre infected with IHHNV by *per os* route and between 27 - 49 d later, they were challenged *per os* with WSSV infected tissues. Shrimp pre-infected with IHHNV showed survival between 25 and 44% compared to 0% in controls inoculated only with WSSV. Survival differences were due to the line of shrimp used, which were crosses of IHHNV susceptible and IHHNV resistant lines, and an IHHNV susceptible line, being this the one with lowest survival. In surviving shrimp, IHHNV viral load was much higher than that of WSSV (10^9^ vs 10^2^ copies), whereas moribund and dead shrimp had higher WSSV load than IHHNV (10^7^ vs 10^4^), respectively. The protective effect of IHHNV infection lasted at least 6 weeks.

Another study evaluated the interference of IHHNV or inactivated WSSV against WSSV in *P. vannamei* postlarvae. Shrimp treated with IHHNV at nauplius V or zoea I stages or treated with formalin-inactivated WSSV showed a delay in shrimp mortality from day 4 until the end of the experiment, indicating an interfering effect of such treatments. Nonetheless, shrimp treated with IHHNV or inactivated WSSV had 4 and 4.7% survival at 10 d post WSSV challenge, respectively. Surviving shrimp had similar virus loads between IHHNV and WSSV (9.0 x 10^1^ - 1.2 x 10^2^ vs 4.4 x 10^2^ - 6.0 x 10^2^, respectively), whereas moribund shrimp had higher WSSV load (1.5 x 10^4^ - 2.3 x 10^4^ vs 2.2 x 10^9^ - 9.0 x 10^8^, respectively) (33).

The interference between IHHNV and WSSV inoculated *per os* in juvenile *P. vannamei* was evaluated at different times after IHHNV inoculation (0 to 50 d). Shrimp challenged only with WSSV died at 3 dpi, whereas shrimp treated with IHHNV between 30 and 50 d before WSSV challenge died at 5 dpi. The virus load in shrimp treated with IHHNV showed an increase during the 50 d peaking at 40 d with an average IHHNV virus load of 2.6 × 10^9^ copies μg^–1^ DNA. Shrimp challenged with WSSV showed a reduction in IHHNV load (2.7 × 10^7^ and 9.7 × 10^7^ copies μg^–1^ DNA) compared to shrimp inoculated only with IHHNV (2.5 × 10^8^ - 2.6 × 10^9^ copies μg^–1^ DNA) at 30 and 40 d, respectively. A delay in mortality due to WSSV infection was a function of IHHNV virus load and duration of infection. A significant delay in mortality occurred when IHHNV load was higher than 10^8^ copies μg^–1^ DNA at the time of WSSV challenge (34).

Another work was done in batches of *P. monodon* juveniles pre-infected with IHHNV and another batch of IHHNV-negative shrimp. Both groups were challenged with WSSV by cohabitation with WSSV-infected shrimp. Results showed significant differences in time of mortality between the two groups: 10.3 ± 2.7 d in the IHHNV-negative group and 14.7 ± 4.9 d in the IHHNV-infected group. Significant differences in WSSV load were found in the two groups: 1.74 × 10^7^ ± 1.78 × 10^7^ copies in the IHHNV-negative group and 2.74 × 10^6^ ± 2.41 × 10^6^ copies in the IHHNV-infected group (35).

An *in vitro* assay was done to determine the interference mechanism of IHHNV and WSSV using a competitive ELISA assay with *P. vannamei* gill cells cultures. Digoxigenin-labeled WSSV and unlabeled IHHNV were used. Gill cell membranes added with unlabeled IHHNV interfered with digoxigenin-labeled WSSV, indicating an interfering effect of IHHNV with WSSV by competition for binding to the cellular membrane. An inverse assay using labeled IHHNV and unlabeled WSSV on gill cell membranes also showed that unlabeled WSSV interfered attachment of labeled IHHNV to cell membranes even in a higher degree. This suggests that WSSV also competes with IHHNV for binding to the cellular membrane (36).

## Discussion

Virus interference is an old and interesting research subject, of which mechanisms in many pairs of viruses are still to be uncovered. The use of modern cell cultures and the development of novel *in vitro* systems to study virus-virus interference may help to unravel such mechanisms. Virus interference might have different mechanisms, of which virus exclusion - either mutual or affecting one of the viruses – may be the most common. This type of interference has been recorded in homologous and heterologous viruses in different vertebrate hosts.

In crustaceans, virus interference is rather a novel phenomenon which so far has been limited by the lack of continuous cell cultures to study virus-host and virus-virus interactions. Efforts to understand the mechanism of interference between pathogenic viruses with less pathogenic ones have been done both *in vivo* (32–35) and *in vitro* (36). Nonetheless, the *in vivo* study of virus interference in these hosts has gained renewed interest as it may provide a new means to control lethal viral pathogens in the absence of an adaptive immune system and lack of effective antiviral treatments. Due to the heavy impact of WSSV outbreaks in farmed shrimp worldwide since the dawn of the XXI century, different methods aimed to curb the disease have been explored. The fact that shrimp previously infected with IHHNV may induce a reduction in severity of WSSV disease and/or mortality, makes this interference a possible natural control method against WSSV. Hence, the phenomenon gained a renewed interest and was investigated between 2003 and 2016, which has been the most recent research trend on the subject. Moreover, WSSV continues to be a major threat to shrimp aquaculture, whereas the Pacific white shrimp appears to have reduced its susceptibility to IHHNV disease (63, 64). Two recent studies showed that these viruses compete by mutual exclusion for cellular receptors (35, 36). Both IHHNV and WSSV produce systemic infections in shrimp, but some differences between the infection and replication steps may influence the outcome of the interaction. WSSV replicates fast, inducing disease and mortality within 3-5 dpi (62). In contrast, IHHNV has a slower replication, which varies from 7-15 dpi and it seldom induces mortality (64). Further, WSSV may use additional cell receptors to enter cells, as it has a broad crustacean host range, in contrast to IHHNV which may enter cells with one of the four polypeptides present on its virion (60). *In vivo* experiments to evaluate interference between these viruses would have to be done at different stages of IHHNV replication and then challenge with WSSV, or inoculating viruses with different doses and/or inoculation routes to determine the best conditions to induce virus interference and higher shrimp survival.

It is possible that the exclusion interference also occurs in the virus pairs reported for crustaceans such as TSV and YHV. These RNA viruses have similar tissue tropism and replicate in the same cell location. A reduced YHV replication and damage was observed in target organs such as lymphoid organ, gill epithelium and foregut of shrimp preinfected with TSV. Although in chronic TSV infection, the virus is not found in many epithelial tissues, still it was able to hinder YHV replication in other target tissues (31).

Research in invertebrate viruses is important because they are often dangerous agents able to cause disease and massive mortalities to many species of economic interest. Nonetheless, they also have potential to be used as control agents of pests and invasive species (65). Further, recent advances in high throughput sequencing has allowed a sharp progress in sequence capacity resulting in increased number of species with complete genome sequences, including viruses. RNA-seq has proved very useful identifying novel viruses in complex or unusual samples. Two main sequence strategies are used for identifying new viral sequences: (i) short read sequencing (i.e. Illumina) which has high throughput and sequencing depth, allowing the identification of a wide range of novel virus sequences or variants of known viruses affecting invertebrates, especially those of economic importance in animal production, affecting species of ecological importance, and/or disease agents in humans (65). This strategy makes it possible to analyze a great number of samples in an economic fashion. (ii) Long reads sequencing is used to decipher incomplete genome assemblies from highly complex samples containing mixed host and pathogen DNA. With these strategies it has been possible to determine whole-genome sequencing of numerous organisms including several previously unknown viruses (65).

The diversity of viral communities can be determined using environmental DNA (eDNA). This method requires the analysis of DNA/RNA obtained from the environment or organisms, including soil, water, host tissues, feces and other samples. Two common eDNA methods are amplicon-based (metabarcoding) or whole shotgun metagenomics/metatranscriptomics. The former amplifies and sequences variable regions of conserved genes (i.e. mitochondrial cytochrome oxidase I and 16S, 18S and 28S ribosomal RNA) or mitochondrial intergenic regions that are shared across a group or sub-groups of a selected organism. The diversity of the resulting sequences are used to assign operational taxonomic units (OTUs). In the case of viruses which lack these conserved regions, the latter method is used to study viral communities in environmental samples (65).

In conclusion, virus interference is an old phenomenon that implies the interaction of two viruses within a host cell, resulting in the inhibition of replication of at least one of the viruses. This phenomenon may have various mechanisms, of which three have been reported. The exclusion interference may be common in viruses infecting animals. Heterologous virus interference between a low and a highly pathogenic viruses has been reported in farmed shrimp and it could be used as a natural control strategy against highly pathogenic viruses. As sequencing technology advances, better knowledge on environmental and crustacean virus diversity will be gained, and also in virus genome organization and gene interactions between host - pathogen and virus - virus interactions, may help elucidate the mechanisms that drive this phenomenon.

## Author Contributions

The author confirms being the sole contributor of this work and has approved it for publication.

## Funding

The present work was supported by Instituto Politecnico Nacional projects SIP20200533 and SIP20210092. COFAA-IPN provided financial support to publish this article.

## Conflict of Interest

The author declares that the research was conducted in the absence of any commercial or financial relationships that could be construed as a potential conflict of interest.
